# Long working hours and dental caries: A 12-year longitudinal study of 1.1 million workers

**DOI:** 10.5271/sjweh.4322

**Published:** 2026-11-01

**Authors:** Min Young Park, Joonho Ahn

**Affiliations:** 1 Department of Occupational and Environmental Medicine, Seoul St. Mary’s Hospital, College of Medicine, The Catholic University of Korea, Seoul, Republic of Korea.; 2 Department of Occupational and Environmental Medicine, Samsung Kangbuk Hospital, Sungkyunkwan University School of Medicine, Seoul, Republic of Korea.

**Keywords:** teeth, tooth decay, dental care, dental cavity, occupational health, oral health, oral hygiene

## Abstract

**Objectives:**

Dental caries is a leading noncommunicable disease worldwide; however, its association with occupational factors, particularly long working hours, has not been elucidated. This study examined the longitudinal association between long working hours and dental caries in a large Korean working-age cohort.

**Methods:**

Using data from the Kangbuk Samsung Health Study (2013–2024), we analyzed 1 136 767 person-visits of workers aged 20–64 years. Long working hours were defined as >52 hours per week. Dental caries was identified from clinical oral examinations performed by licensed dentists. Associations were estimated using generalized estimating equations adjusted for age, gender, and educational level. Subgroup and sensitivity analyses were also performed.

**Results:**

Long working hours were significantly associated with dental caries (odds ratio 1.024, 95% confidence interval 1.007–1.041). In the subgroup analyses, compared with their respective counterparts, the association between long working hours and dental caries was significantly stronger among women, individuals with a college-level education or higher, and those with proactive oral health behaviors such as dental visits and toothbrushing before sleep.

**Conclusions:**

Long working hours were independently associated with the risk of dental caries; women and highly educated workers exhibited a stronger association with dental caries. These findings suggest the potential value of integrating oral health strategies into occupational health policies, particularly among more vulnerable subgroups.

Dental caries is one of the most prevalent noncommunicable diseases worldwide and is estimated to affect over 2 billion people with permanent dentition (1). Beyond its substantial individual burden including pain, tooth loss, and impaired quality of life, dental caries imposes serious health and economic burdens on individuals, families, and health systems worldwide, which undermine work productivity and widen social inequalities (2). The etiology of dental caries is multifactorial and involves microbial-, dietary-, and host-related factors, and identifying modifiable risk factors remains a public health priority (3).

Long working hours are recognized as a major occupational health hazard associated with an increased risks for cardiovascular diseases, stroke, and mental disorders (4, 5). Chronic psychosocial stress, sleep disruption, and cascading deterioration of health behaviors, including poor diet, physical inactivity, and reduced engagement in preventive healthcare, can result from long working hours (6). These behavioral consequences are particularly relevant to oral health because dietary patterns, oral hygiene practices, and dental care utilization are among the most stressful and time-sensitive health behaviors (7, 8).

Several studies have explored the link between occupational work conditions and oral health; they suggest that long working hours adversely affect dental health behaviors and oral health outcomes (7–9). However, the existing evidence has been obtained from predominantly cross-sectional designs or panel analyses with limited follow-ups and has focused on outcomes other than dental caries. This gap is particularly noteworthy given that the behavioral and biological mechanisms underlying such an association are plausible. Behaviorally, heavy work demands may compromise oral self-care practices; biologically, chronic work-related stress may adversely affect the oral environment through neuroendocrine pathways (7, 10). Whether these mechanisms translate into a longitudinal increase in caries risk has not been clarified. Moreover, it remains unknown whether this association varies across subgroups. Biological differences in salivary function may render women more susceptible to oral deterioration (11), socioeconomic disadvantage is associated with a higher caries burden (12), oral health behaviors such as dental visits or toothbrushing may further modify the association (7, 8), and health conditions such as diabetes mellitus and behavioral factors such as smoking and obesity may also modulate this relationship (13).

To address this knowledge gap, the present study examined the longitudinal association between long working hours and dental caries in a large Korean working-age cohort. Given the high prevalence of long working hours in the Republic of Korea and the substantial global burden of dental caries, the findings of this study may provide important evidence for integrating oral health into occupational health policies and health promotion in the workplace.

## Methods

### Data source and study participants

The data were obtained from the Kangbuk Samsung Health Study, a robust longitudinal cohort of Korean adults who underwent comprehensive health screenings (14). These screenings were conducted at the Kangbuk Samsung Hospital Total Healthcare Center clinics in Seoul and Suwon, Republic of Korea. The study population comprised individuals who participated in annual or biennial clinical examinations.

Figure 1 illustrates the selection process for the study participants that occurred between 2013 and 2024. From this pool, we applied several exclusion criteria in a stepwise manner to ensure the validity of the analytical sample. We excluded 87 730 person-visits, aged <20 or >64 years. Subsequently, we removed 766 562 non-workers, 437 684 person-visits with missing employment data, and 1398 person-visits employed in the military, agriculture, forestry, and fishery sectors. To maintain the reliability of the exposure variables, we excluded 24 123 person-visits as outliers with extreme working hours, and 252 832 person-visits with <20 working hours per week. An additional 24 636 person-visits with missing covariates and 167 889 person-visits who lacked follow-up measures were excluded. After these systematic exclusions, the final study population contained 1 136 767 person-visits. The annual distributions of the final sample remained consistent throughout the study period, ranging from 33 791 person-visits in 2013 to 130 220 person-visits in 2024.

**Figure 1:**
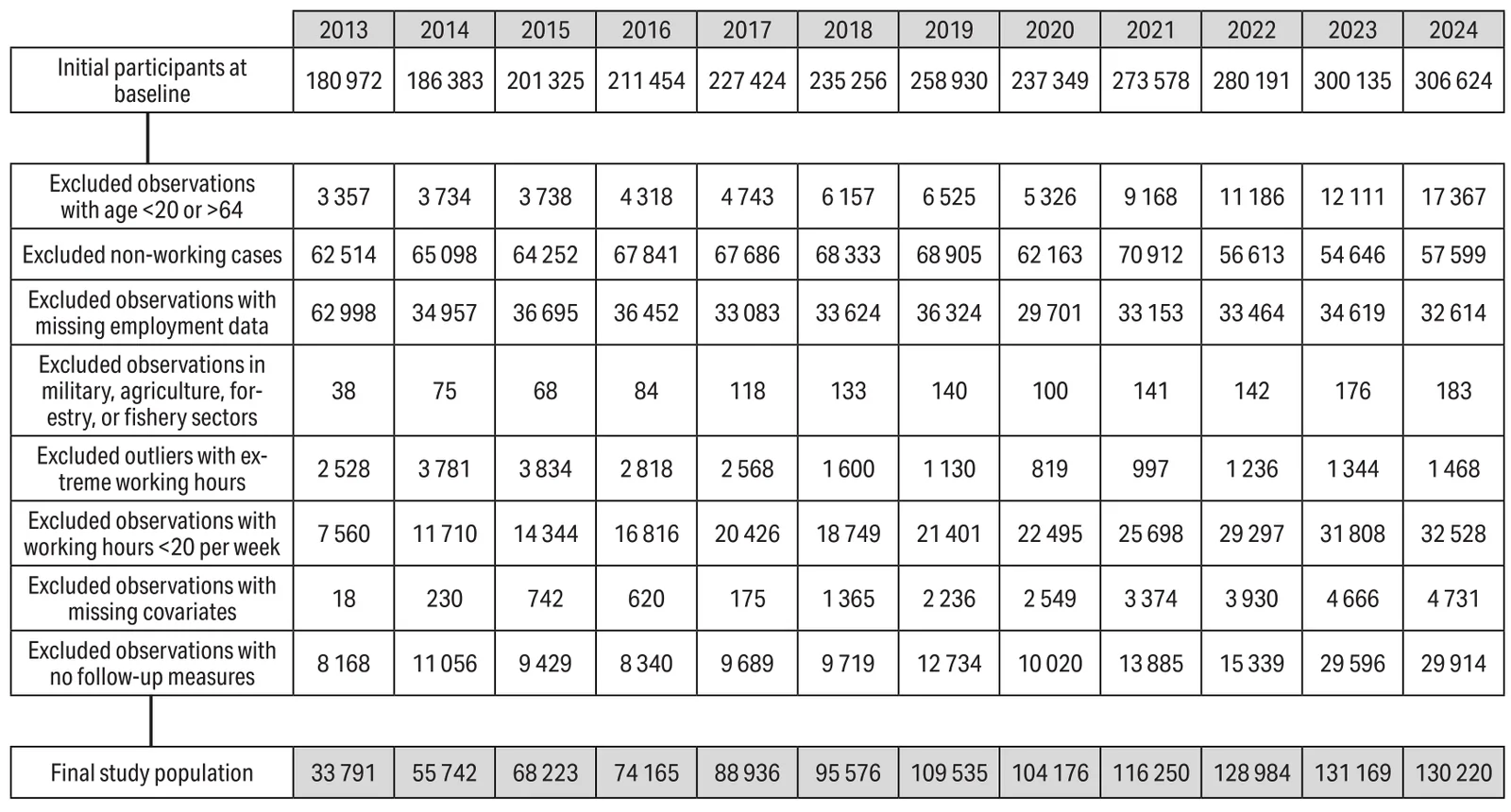
Flow of participant selection.

### Exposure and outcome definitions

The primary exposure was weekly working hours, which were assessed using a self-administered questionnaire. Participants were asked to report, “On average, how many hours per week have you worked at your job, including overtime, during the past year?”. Long working hours were defined as >52 hours per week, a threshold established considering the statutory labor regulations for overwork in the Republic of Korea. person-visits working ≤52 hours were classified into the reference group (15).

The outcome of interest was the presence of dental caries, identified in clinical oral examinations conducted by licensed dentists during the health screenings. Dental caries was defined as the presence of at least one decayed tooth at the time of examination.

### Other variables

Various demographic, socioeconomic, and lifestyle factors were included in this study. Age was categorized into two groups: 20–39 and 40–64 years. Socioeconomic status was assessed based on the highest level of education attained, which was classified as either “high school or lower” or “college or higher.” Oral health behaviors were evaluated based on dental visits within the previous year and tooth brushing frequency before sleep, with the latter categorized as frequent or infrequent. Lifestyle and health-related factors included current smoking and obesity status. Obesity was defined using the body mass index (BMI); individuals with a BMI ≥25 kg/m^2^ were classified as having obesity, and those with a BMI <25 kg/m^2^ were classified as non-obese. Diabetes mellitus was defined based on a combination of clinical data and medical history. The participants were identified as having diabetes mellitus if they met at least one of the following criteria: a glycated hemoglobin level ≥6.5%, self-reported history of diabetes, or current use of glucose-lowering medications.

### Statistical analyses

Statistical analyses were performed using generalized estimating equations (GEE) to account for repeated measurements within individuals. Because the primary outcome was a binary variable (presence of dental caries), the GEE models were fitted using a binomial distribution and a logit link function. An exchangeable working correlation structure was specified to model the intra-subject correlations across multiple visits. Based on the directed acyclic graph illustrating the hypothesized causal pathways between long working hours and dental caries (supplementary material, www.sjweh.fi/article/4322, figure S1), the final adjusted models included age, gender, and socioeconomic status (operationalized as educational level) as covariates.

To evaluate potential effect modifications, subgroup analyses were conducted by stratifying the study population according to age group, gender, education level, dental visits within the previous year, tooth brushing frequency before sleep, diabetes mellitus, current smoking status, and obesity status. The P value for interaction was calculated by including an interaction term between long working hours and each stratification variable in the GEE model and was assessed using the Wald test.

Four sensitivity analyses were performed to assess the robustness of our findings. Although the main analysis excluded person-visits who worked <20 hours per week to focus on a stable working population, the first sensitivity analysis included all individuals regardless of their weekly working hours. The second sensitivity analysis further restricted the study population to individuals who worked ≥35 hours per week. Furthermore, we assessed whether the association remained consistent under different definitions of exposure by redefining long working hours using a lower threshold of >50 hours per week for the third sensitivity analysis and a higher threshold of >54 hours per week for the fourth sensitivity analysis, both of which were compared with the main analysis cutoff of 52 hours.

All statistical analyses were conducted using R (version 4.4.3; R Foundation, Vienna, Austria) and Stata/SE (version 19.0, StataCorp. LLC, College Station, TX, USA). Two-sided P-values <0.05 were considered statistically significant.

### Ethics statement

The study protocol was reviewed and approved by the Institutional Review Board of the Kangbuk Samsung Hospital (IRB No. KBSMC IRB 2025-09-046), and the requirement for informed consent was waived owing to the use of de-identified data.

## Results

The study population general characteristics during the 12-year study period are summarized in table 1. The final analytical sample comprises 1 136 767 person-visits, with the annual number increasing from 33 791 in 2013 to 130 220 in 2024. Throughout the study period, the proportion of participants aged 40–64 years steadily increased, ranging from 50.8% to 75.6%. The majority of the study population were men (70.2–77.3%) with educational attainment of college or higher (87.9–91.7%). The prevalence of long working hours significantly decreased over time, falling from 23.0% in 2013 to 4.1% in 2024. The prevalence of dental caries showed a modest downward trend, decreasing from approximately 24% to 15%, during the study period.

**Table 1:** Characteristics of the study population by year

	2013	2014	2015	2016	2017	2018	2019	2020	2021	2022	2023	2024
	N person visits (%)	N person visits (%)	N person visits (%)	N person visits (%)	N person visits (%)	N person visits (%)	N person visits (%)	N person visits (%)	N person visits (%)	N person visits (%)	N person visits (%)	N person visits (%)
Total	33 791 (100.0)	55 742 (100.0)	68 223 (100.0)	74 165 (100.0)	88 936 (100.0)	95 576 (100.0)	109 535 (100.0)	104 176 (100.0)	116 250 (100.0)	128 984 (100.0)	131 169 (100.0)	130 220 (100.0)
Age group												
20–39	16 632 (49.2)	26 480 (47.5)	33 082 (48.5)	33 335 (44.9)	41 075 (46.2)	39 595 (41.4)	45 102 (41.2)	37 038 (35.6)	41 127 (35.4)	39 468 (30.6)	35 984 (27.4)	31 794 (24.4)
40–64	17 159 (50.8)	29 262 (52.5)	35 141 (51.5)	40 830 (55.1)	47 861 (53.8)	55 981 (58.6)	64 433 (58.8)	67 138 (64.4)	75 123 (64.6)	89 516 (69.4)	95 185 (72.6)	98 426 (75.6)
Sex												
Male	25 587 (75.7)	43 090 (77.3)	52 293 (76.7)	55 755 (75.2)	65 129 (73.2)	69 844 (73.1)	78 757 (71.9)	74 311 (71.3)	82 149 (70.7)	91 194 (70.7)	92 215 (70.3)	91 413 (70.2)
Female	8204 (24.3)	12 652 (22.7)	15 930 (23.3)	18 410 (24.8)	23 807 (26.8)	25 732 (26.9)	30 778 (28.1)	29 865 (28.7)	34 101 (29.3)	37 790 (29.3)	38 954 (29.7)	38 807 (29.8)
Education level												
College or higher	29 713 (87.9)	50 345 (90.3)	61 579 (90.3)	67 461 (91.0)	80 587 (90.6)	87 414 (91.5)	99 180 (90.5)	95 225 (91.4)	106 493 (91.6)	117 962 (91.5)	119 342 (91.0)	119 464 (91.7)
High school or lower	4078 (12.1)	5397 (9.7)	6644 (9.7)	6704 (9.0)	8349 (9.4)	8162 (8.5)	10 355 (9.5)	8951 (8.6)	9757 (8.4)	11 022 (8.5)	11 827 (9.0)	10 756 (8.3)
Long working hours												
No	26 016 (77.0)	42 998 (77.1)	53 392 (78.3)	62 197 (83.9)	77 679 (87.3)	88 189 (92.3)	105 047 (95.9)	100 693 (96.7)	111 944 (96.3)	124 124 (96.2)	125 802 (95.9)	124 897 (95.9)
Yes	7775 (23.0)	12744 (22.9)	14 831 (21.7)	11 968 (16.1)	11 257 (12.7)	7387 (7.7)	4488 (4.1)	3483 (3.3)	4306 (3.7)	4860 (3.8)	5367 (4.1)	5323 (4.1)
Dental caries												
No	26 219 (77.6)	44 386 (79.6)	52 774 (77.4)	57 958 (78.1)	67 400 (75.8)	74 399 (77.8)	86 118 (78.6)	84 220 (80.8)	94 563 (81.3)	107 530 (83.4)	111 494 (85.0)	110 442 (84.8)
Yes	7572 (22.4)	11 356 (20.4)	15 449 (22.6)	16 207 (21.9)	21 536 (24.2)	21 177 (22.2)	23 417 (21.4)	19 956 (19.2)	21 687 (18.7)	21 454 (16.6)	19 675 (15.0)	19 778 (15.2)
Dental visit within the past year												
No	15 590 (46.1)	25 712 (46.1)	36 142 (53.0)	40 961 (55.2)	47 690 (53.6)	50 774 (53.1)	57 271 (52.3)	55 950 (53.7)	62 723 (54.0)	68 427 (53.1)	55 894 (42.6)	53 081 (40.8)
Yes	17 843 (52.8)	30 007 (53.8)	32 069 (47.0)	33 178 (44.7)	41 244 (46.4)	44 788 (46.9)	52 259 (47.7)	48 224 (46.3)	53 522 (46.0)	60 554 (46.9)	75 272 (57.4)	77 139 (59.2)
Missing	358 (1.1)	23 (0.0)	12 (0.0)	26 (0.0)	2 (0.0)	14 (0.0)	5 (0.0)	2 (0.0)	5 (0.0)	3 (0.0)	3 (0.0)	0 (0.0)
Toothbrushing before sleep												
Infrequent	6712 (19.9)	12 175 (21.8)	14 591 (21.4)	14 768 (19.9)	16 514 (18.6)	16 929 (17.7)	18 393 (16.8)	16 471 (15.8)	17 590 (15.1)	18 898 (14.7)	19 005 (14.5)	18 056 (13.9)
Frequent	24 950 (73.8)	43 529 (78.1)	53 616 (78.6)	59 380 (80.1)	72 418 (81.4)	78 630 (82.3)	91 137 (83.2)	87 702 (84.2)	98 654 (84.9)	110 082 (85.3)	112 161 (85.5)	112 163 (86.1)
Missing	2129 (6.3)	38 (0.1)	16 (0.0)	17 (0.0)	4 (0.0)	17 (0.0)	5 (0.0)	3 (0.0)	6 (0.0)	4 (0.0)	3 (0.0)	1 (0.0)
Diabetes mellitus												
No	32 392 (95.9)	53 542 (96.1)	65 574 (96.1)	71 136 (95.9)	85 472 (96.1)	91 280 (95.5)	103 485 (94.5)	98 925 (95.0)	110 045 (94.7)	121 325 (94.1)	123 030 (93.8)	121 169 (93.0)
Yes	1385 (4.1)	2180 (3.9)	2633 (3.9)	3006 (4.1)	3448 (3.9)	4282 (4.5)	6041 (5.5)	5198 (5.0)	6190 (5.3)	7357 (5.7)	7673 (5.8)	7910 (6.1)
Missing	14 (0.0)	20 (0.0)	16 (0.0)	23 (0.0)	16 (0.0)	14 (0.0)	9 (0.0)	53 (0.1)	15 (0.0)	302 (0.2)	466 (0.4)	1141 (0.9)
Current smoking status												
No	24 888 (73.7)	41 719 (74.8)	53 381 (78.2)	58 668 (79.1)	71 869 (80.8)	77 841 (81.4)	89 592 (81.8)	86 597 (83.1)	96 763 (83.2)	106 429 (82.5)	107 688 (82.1)	1074 42 (82.5)
Yes	8824 (26.1)	13 317 (23.9)	14 773 (21.7)	15 463 (20.8)	17 052 (19.2)	17 730 (18.6)	19 854 (18.1)	17 579 (16.9)	19 486 (16.8)	22 555 (17.5)	23 480 (17.9)	22 778 (17.5)
Missing	79 (0.2)	706 (1.3)	69 (0.1)	34 (0.0)	15 (0.0)	5 (0.0)	89 (0.1)	0(0.0)	1 (0.0)	0 (0.0)	1 (0.0)	0 (0.0)
Obesity												
No	21 850 (64.7)	36 402 (65.3)	43 135 (63.2)	46 325 (62.5)	55 593 (62.5)	60 079 (62.9)	68 327 (62.4)	63 971 (61.4)	71 024 (61.1)	78 652 (61.0)	79 240 (60.4)	78 127 (60.0)
Yes	11 941 (35.3)	19 340 (34.7)	25 088 (36.8)	27 840 (37.5)	33 343 (37.5)	35 497 (37.1)	41 208 (37.6)	40 205 (38.6)	45 226 (38.9)	50 332 (39.0)	51 929 (39.6)	52 093 (40.0)

In the main analysis, the total adjusted model showed that long working hours were significantly associated with an increased risk for dental caries [odds ratio (OR) 1.024, 95% confidence interval (CI) 1.007–1.041]; this result was consistently observed across all sensitivity analyses (table 2, supplementary table S1). The stratified analyses revealed significant effect modification by gender in both the main analysis (P for interaction=0.0025) and all sensitivity analyses (table 2, supplementary table S1). The association was consistently more pronounced for women (OR 1.079, 95% CI 1.029–1.131) than men (OR 1.021, 95% CI 1.003–1.039). Age group did not show a significant interaction in the main analysis, and the relative magnitude of associations varied across sensitivity analyses without a consistent pattern favoring either age group. Regarding health behaviors and underlying conditions, obesity and current smoking status had statistically significant interactions in the main analysis; however, the direction and magnitude of the associations were not consistent across sensitivity analyses as different subgroups showed stronger associations depending on the sensitivity analyses. No significant interaction was observed for diabetes mellitus in the main analysis, and this pattern remained most consistent across sensitivity analyses. Consistent patterns were observed in both main and sensitivity analyses for oral health-related factors. The association between long working hours and dental caries was stronger among individuals who had visited a dentist within the previous year (OR 1.048, 95% CI 1.022–1.075) compared with those who had not (OR 1.009, 95% CI 0.987–1.031; P for interaction=0.0031). Similarly, the participants who reported frequent tooth brushing before sleep had a stronger association (OR 1.027, 95% CI 1.008–1.046) than those who reported infrequent tooth brushing (OR 0.998, 95% CI 0.963–1.035; P for interaction=0.033). Educational level also had a consistent effect modification; stronger associations were observed among individuals with a college-level education or higher (OR 1.082, 95% CI 1.063–1.101) than among those with a high school education or lower (OR 1.018, 95% CI 0.961–1.079; P for interaction=0.0256). These patterns were consistently observed across all the sensitivity analyses.

**Table 2:** Association between long working hours and dental caries. [OR=odds ratio; CI=confidence interval.]

	OR (95% CI) ^a^	P for interaction ^d^
Total (crude)	1.183 (1.164–1.202)	
Total (adjusted) ^b, c^	1.024 (1.007–1.041)	
Age group		
20–39	1.034 (1.010–1.059)	0.6362
40–64	0.998 (0.975–1.021)	
Sex		
Male	1.021 (1.003–1.039)	0.0025
Female	1.079 (1.029–1.131)	
Education level		
High school or lower	1.018 (0.961–1.079)	0.0256
College or higher	1.082 (1.063–1.101)	
Dental visit within the past year		
No	1.009 (0.987–1.031)	0.0031
Yes	1.048 (1.022–1.075)	
Toothbrushing before sleep		
Infrequent	0.998 (0.963–1.035)	0.0330
Frequent	1.027 (1.008–1.046)	
Diabetes mellitus		
No	1.026 (1.009–1.043)	0.5568
Yes	0.962 (0.889–1.041)	
Current smoking status		
No	1.021 (1.001–1.041)	0.0800
Yes	1.020 (0.988–1.052)	
Obesity		
No	1.036 (1.014–1.059)	0.0086
Yes	1.014 (0.989–1.040)	

^a^ Estimated using generalized estimating equations (GEE). ^b^ The adjusted model included age, sex, and educational level. ^c^ For stratified analyses, the stratification variable was excluded from the corresponding adjusted model. ^d^ Calculated by including an interaction term between long working hours and each stratification variable in the GEE model, assessed using a Wald test.

## Discussion

In this large longitudinal cohort study with over 1 million Korean workers followed for 12 years, long working hours were independently associated with a significantly increased risk of dental caries after adjusting for age, gender, and educational level, with consistent findings across the four sensitivity analyses. These results indicated a robust longitudinal relationship between long working hours and dental caries, which to our knowledge is the first study to provide this evidence. Thus, these findings complement and strengthen prior research that was limited to cross-sectional and short-term panel designs.

Previous studies have suggested an association between heavy work schedules and poor dental outcomes. In Japan, a study reported that male financial workers with the highest overtime hours (>80 hours/month) had more than three times the odds of untreated tooth decay compared to those without overtime; “being too busy with work” was cited as the most common reason for leaving caries untreated (9). Another study conducted in Japan found that overtime was associated with a reduced frequency of preventive dental visits, particularly among male workers in their 50s (16). In the Republic of Korea, a cross-sectional analysis using national survey data identified long working hours as a significant risk factor for periodontitis in a dose–response manner (17), and a panel study found that workers who exceeded 52 hours per week reported significantly more unmet dental care needs attributable to time constraints (8). Consistent with these findings, the present study extended this evidence by directly examining clinically verified dental caries, rather than dental service utilization or periodontal outcomes, in a large longitudinal cohort with repeated measures; thereby offer a more comprehensive characterization of the relationship between long working hours and oral health.

The biological and behavioral mechanisms underlying this association are plausible and multifold. A nationwide Korean study reported that long working hours are associated with reduced adherence to all three major preventive oral health behaviors (dental check-ups, tooth brushing frequency, and interdental cleaning) for both sexes, which provides a direct behavioral association of long working hours with dental caries risk (7). Long working hours disrupt dietary patterns; workers with heavy time demands report a higher consumption of sugary beverages and high-fat, high-sugar snacks while reducing regular meal intake (18, 19). In addition to these behavioral factors, chronic psychosocial stress associated with long working hours may adversely affect the oral environment via neuroendocrine pathways. Activation of the hypothalamic-pituitary-adrenal axis under sustained work-related stress elevates cortisol levels, which can suppress salivary flow rate, alter salivary composition, reduce buffering capacity, increase oral acidity, and create an environment conducive to cariogenic bacteria proliferation (10, 20–22).

Beyond these behavioral and biological pathways, structural barriers to dental care may further contribute. In the Republic of Korea, out-of-pocket costs for dental services substantially exceed the OECD average (84.2% versus 55.1% of total dental expenditure) (23), suggesting that workers with long working hours may face compounded barriers of time scarcity and financial burden that limit access to timely preventive dental care.

The subgroup analyses revealed several noteworthy patterns of effect modification. The association between long working hours and dental caries was significantly stronger among women than men, which was consistent across all sensitivity analyses. This sex difference is plausible through both biological and social mechanisms. Biologically, women generally exhibit lower salivary pH, reduced salivary flow rates, and different salivary buffering capacities than those of men, which are partly attributable to hormonal fluctuations during the menstrual cycle, pregnancy, and menopause that collectively render the oral environment more cariogenic (11, 24, 25). Consequently, further stress-induced suppression of salivary function from long working hours may disproportionately affect women who already operate with reduced salivary protective capacity. Socially, women in paid employment often bear a disproportionate share of unpaid domestic labor and caregiving responsibilities, a phenomenon described as the double burden of paid and unpaid work (26). Such time constraints may reduce opportunities for preventive dental care and oral self-care (16, 27), representing an additional pathway through which long working hours may more severely affect women’s oral health. Although the absolute difference in OR between women and men (OR 1.079 versus 1.021) may appear modest at the individual level, long working hours remain highly prevalent in many countries worldwide; even a small incremental risk per person may translate into a substantial number of preventable caries cases at the population level.

The patterns of effect modification observed across oral health behaviors and educational levels shared a common interpretive framework. The workers with poor oral hygiene behaviors or lower socioeconomic status already have a substantially elevated baseline caries burden (12), thus, the incremental contribution of long working hours may be attenuated by a ceiling effect. Conversely, among those with proactive oral health behaviors, frequent dental visits increase the likelihood of detecting subclinical caries, which may amplify the observed association through greater clinical ascertainment. A similar mechanism may operate among highly educated workers. Evidence consistently indicates that higher educational attainment is associated with greater utilization of preventive dental services (28), increasing the probability of detecting caries lesions that might otherwise remain undiagnosed. Furthermore, the cognitive demands and psychological pressure inherent in professional occupations may compound the cariogenic stress burden of long working hours. While the difference in odds ratios between educational groups (college or higher: OR 1.082 versus high school or lower: OR 1.018) may appear limited in magnitude, nearly 90% of the study population had attained college-level education or higher; the population-level implications of this association are therefore not negligible. Although these explanations remain speculative in the absence of mediation analyses, they are mechanistically plausible and warrant further investigation in future studies incorporating more detailed data on dental care utilization, out-of-pocket costs, and other potential mediators.

This study has several strengths to consider. The large sample size (1 136 767 person-observations), 12-year follow-up, clinically verified caries outcomes by licensed dentists, and GEE-based repeated measures analyses collectively enhanced the internal validity and statistical power of the findings.

This study also has several limitations. First, despite adjusting for age, sex, and educational level based on a directed acyclic graph, residual confounding from unmeasured variables such as dietary sugar intake, fluoride exposure, and oral hygiene product use, must be acknowledged. Future studies incorporating detailed dietary and behavioral assessments would strengthen causal inferences. Second, several potential biases warrant consideration. Excluded individuals were more likely to be female and less educated (supplementary table S2), suggesting a disproportionate exclusion of vulnerable populations who typically face higher oral health risks; their exclusion likely attenuated the observed estimates, and caution is warranted when generalizing the findings beyond the stable, highly educated workforce represented in this study. Working hours were self-reported, and while asking about average hours over the past year is a standardized approach in large-scale national surveys, recall bias cannot be entirely excluded. Additionally, workers who continued working long hours in later years may represent an increasingly selected subgroup concentrated in occupational sectors or circumstances less amenable to regulatory compliance. The heightened job demands characteristic of such a subgroup may independently elevate caries risk beyond the effect of working hours per se, potentially introducing a degree of upward bias in the estimated associations during the later study period. Conversely, the progressive aging of this group over time may have acted as a counterbalancing downward bias given the weaker association observed among older workers. Third, the clinical examination data did not allow characterization of caries severity, affected tooth surfaces, or lesion progression rates, which precluded a more nuanced dose–response analysis. Finally, the dramatic decline in the prevalence of long working hours observed over the study period, from 23.0% in 2013 to 4.1% in 2024, likely reflected the Republic of Korea regulatory reforms in working hour legislation, which may have introduced period effects difficult to isolate fully from secular trends in oral health behaviors. However, because GEE estimates the association based on within-individual repeated measures, any period effects that applied uniformly across the population were unlikely to have substantially biased comparisons. Furthermore, the GEE model adjusted for age, sex, and educational level, mathematically controlling for these macroscopic compositional shifts; the fact that a statistically significant association remained despite this strong downward pressure further underscores the robustness of the findings.

In conclusion, this large longitudinal cohort study provides evidence that long working hours are associated with an increased risk of dental caries among Korean workers. Notably, stronger associations were observed among women, highly educated individuals, and those with proactive oral health behaviors. While the modest effect size observed at the individual level warrants cautious interpretation, the widespread prevalence of long working hours globally suggests that the population-level burden may not be negligible. These findings suggest the potential value of incorporating oral health programs into occupational health surveillance and workplace health promotion, particularly among more vulnerable subgroups.

## Supplementary material

Supplementary material 1 Supplementary material

## Data Availability

Data are not publicly available. The datasets analyzed during the current study were sourced from the Kangbuk Samsung Health Study. Access to these data is restricted and is available only to authorized researchers within a secure research environment in accordance with institutional data protection policies.
