# Supplementary material for: Long working hours and dental caries: A 12-year longitudinal study of 1.1 million workers
Source: Scand J Work Environ Health. 2026 Oct 26;52(6):672–9. doi: 10.5271/sjweh.4322 (PMC13733357; doi:10.5271/sjweh.4322)
Supplement: Supplementary file 1 [file SJWEH-52-6-672-s001.pdf]

Long working hours and dental caries: A 12-year longitudinal study of 1.1 million workers[1]

by Min Young Park, PhD, Joonho Ahn, PhD [2]

1. Supplementary material
2. Correspondence to: Joonho Ahn, Department of Occupational and Environmental Medicine, Kangbuk Samsung Hospital Sungkyunkwan University School of Medicine, 29 Saemunan-ro, Jongno-gu, Seoul 03181, Republic of Korea. [E-mail: drcox@naver.com].
ORCiDs Min Young Park: 0000-0002-7812-7912 Joonho Ahn: 0000-0002-5940-9877

Supplementary Figure S1. Directed acyclic graph (DAG) illustrating the hypothesized causal pathways between long working hours and dental caries

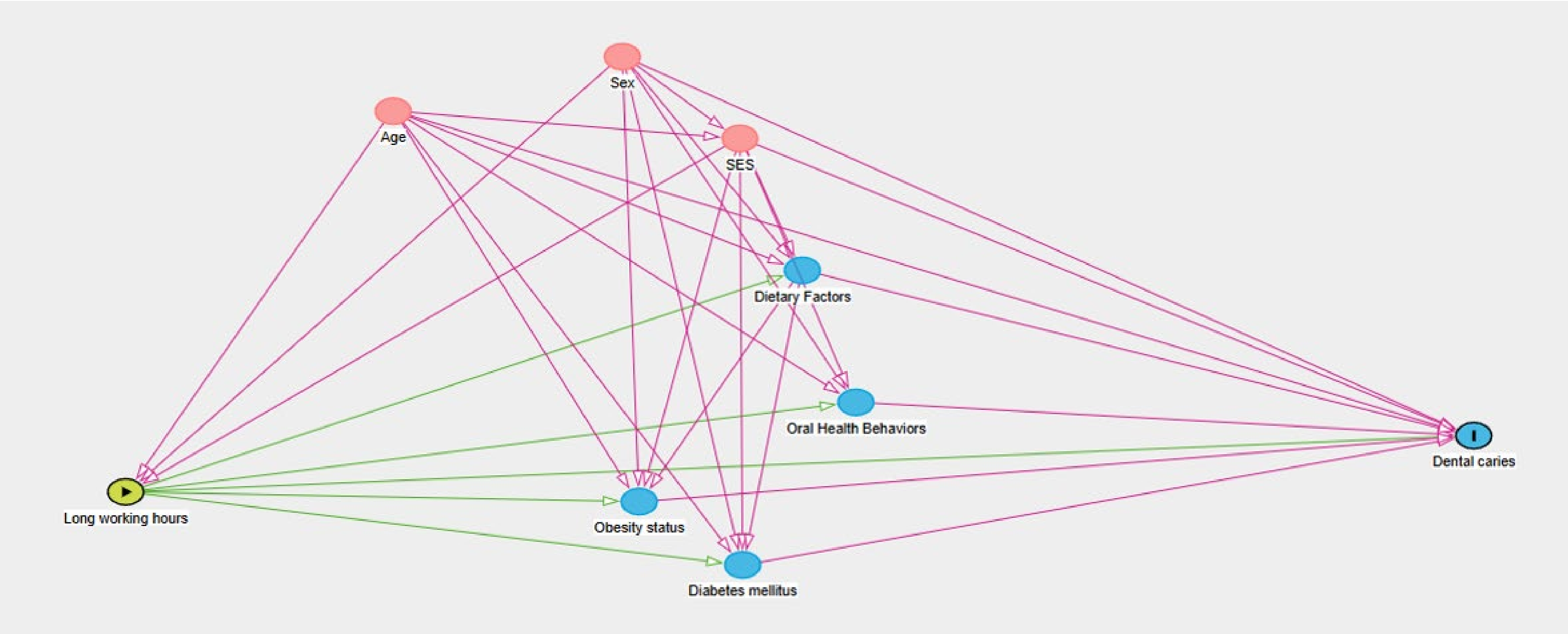

Supplementary Table S1. Sensitivity analyses for the association between long working hours and dental caries

	Sensitivity analysis 1		Sensitivity analysis 2		Sensitivity analysis 3		Sensitivity analysis 4	
	OR (95% CI)	P for interaction	OR (95% CI)	P for interaction	OR (95% CI)	P for interaction	OR (95% CI)	P for interaction
Total (crude)	1.181 (1.163–1.200)		1.170 (1.152–1.189)		1.131 (1.117–1.145)		1.185 (1.166–1.205)	
Total (adjusted)	1.021 (1.004–1.037)		1.021 (1.005–1.038)		1.049 (1.028–1.069)		1.035 (1.011–1.060)	
Age group								
20-39	1.032 (1.008–1.056)	0.8620	1.030 (1.006–1.055)	0.4985	1.022 (1.004–1.040)	0.0091	0.997 (0.974–1.021)	0.5434
40-64	0.997 (0.974–1.020)		0.994 (0.972–1.018)		1.077 (1.018–1.138)		1.099 (1.028–1.175)	
Sex								
Male	1.018 (1.000–1.035)	0.0023	1.020 (1.002–1.038)	0.0315	1.025 (1.011–1.039)	0.0214	1.020 (1.002–1.039)	0.0008
Female	1.075 (1.026–1.127)		1.058 (1.010–1.109)		1.140 (1.099–1.183)		1.091 (1.039–1.145)	
Education level								
High school or lower	1.011 (0.955–1.069)	0.0159	1.006 (0.950–1.065)	0.0160	1.073 (1.028–1.120)	0.0077	1.027 (0.968–1.090)	0.0540
College or higher	1.077 (1.059–1.096)		1.074 (1.055–1.092)		1.090 (1.074–1.105)		1.083 (1.064–1.103)	
Dental visit within the past year								
No	1.010 (0.988–1.032)	0.0046	1.006 (0.984–1.028)	0.0025	1.020 (1.002–1.037)	0.0089	1.007 (0.985–1.030)	0.0014
Yes	1.046 (1.020–1.072)		1.043 (1.017–1.070)		1.070 (1.049–1.091)		1.052 (1.025–1.079)	
Toothbrushing before sleep								
Infrequent	0.994 (0.960–1.029)	0.0196	0.996 (0.961–1.032)	0.0558	1.015 (0.986–1.045)	0.0149	1.003 (0.967–1.041)	0.0552
Frequent	1.024 (1.005–1.043)		1.023 (1.005–1.043)		1.044 (1.028–1.059)		1.027 (1.007–1.046)	
Diabetes mellitus								
No	1.022 (1.005–1.039)	0.9079	1.023 (1.006–1.040)	0.6114	1.039 (1.025–1.053)	0.0071	1.027 (1.009–1.044)	0.6286
Yes	0.978 (0.905–1.056)		0.958 (0.886–1.037)		0.989 (0.934–1.047)		0.969 (0.893–1.050)	
Current smoking status								
No	1.015 (0.996–1.035)	0.2291	1.017 (0.998–1.037)	0.1934	1.046 (1.030–1.061)	0.0080	1.023 (1.002–1.043)	0.0538
Yes	1.026 (0.995–1.057)		1.017 (0.986–1.049)		1.005 (0.979–1.031)		1.018 (0.986–1.050)	
Obesity								

No	1.033 (1.011–1.055)	0.0123	1.032 (1.009–1.055)	0.0292	1.025 (1.005–1.045)	0.0101	1.011 (0.985–1.037)	0.0022
Yes	1.013 (0.989–1.038)		1.013 (0.988–1.039)		1.038 (1.023–1.053)		1.026 (1.007–1.045)	

Notes.

Odds ratios (ORs) and 95% confidence intervals (CIs) were estimated using generalized estimating equations (GEE).

The adjusted model included age, sex, and educational level.

For stratified analyses, the stratification variable was excluded from the corresponding adjusted model.

P for interaction was calculated by including an interaction term between long working hours and each stratification variable in the GEE model and was assessed using a Wald test

Sensitivity analyses were performed by varying the study population and the definition of long working hours: Sensitivity analysis 1 included all individuals regardless of weekly working hours; Sensitivity analysis 2 restricted the study population to individuals working at least 35 hours per week; Sensitivity analysis 3 involved redefining long working hours using a lower threshold of more than 50 hours per week, instead of the main analysis cutoff of 52 hours; and sensitivity analysis 4 involved redefining long working hours using a higher threshold of more than 54 hours per week, instead of the main analysis cutoff of 52 hours.

Supplementary Table S2. Comparison of demographic characteristics between included and excluded participants

	Included participants<br>N (%)	Excluded participants<br>N (%)
Total	1136767 (100.0)	908562 (100.0)
Age group		
20-39	420715 (37.0)	360753 (39.7)
40-64	716055 (63.0)	547809 (60.3)
Sex		
Male	821737 (72.3)	518502 (57.1)
Female	315030 (27.7)	390060 (42.9)
Educational level		
College or higher	1034765 (91.0)	715532 (78.8)
High school or lower	102002 (9.0)	154748 (17.0)
Missing	0 (0.0)	38282 (4.2)

Note. Values are presented as number of person-visits (%).
